# The Retractive Power of the Bowels Considered in Medical Practice

**Published:** 1884-12

**Authors:** Frank H. Nichols

**Affiliations:** Atlanta, Ga.


### THE RETRACTIVE POWER OF THE BOWELS CONSIDERED IN MEDICAL PRACTICE.

By FRANK H. NICHOLS. M. D., ATLANTA, GA.

In a former communication we briefly called the attention of medical men to the important fact that there was a real, positive retractive power inherent in the bowels; that this power exists in health, and was capable of being excited at the will of the surgeon in all cases of hernia; that its action was salutary, and, with proper taxis, always successful in strangulated hernia—aiding the surgeon and aiding nature in the only safe mode of recovery known to the profession. We wish to emphasize these statements, because we know them to be true, and susceptible of proof by clinical demonstration.

If we examine the annual reports from the leading hospitals in Europe or America, we find a fearful mortality attending surgical operations for the relief of strangulated hernia. Drs. Gross and Hamilton have published to the profession the result of their practice. They both lost one-third of their patients. This great mortality gives to this ailment a portentous dread that extends justly to others.

In practice we mainly rely upon this retractive power of the bowels. We find it always an efficient aid in reduction, pulling back the protruding part. After the reduction, there being no solution of continuity of tissue by the surgeon's knife; no exposure of congested or inflamed parts to the open air, the recovering power of nature within speedily repairs the damage done to the body. Our experience leads us to believe that no internal medicine is really necessary in strangulated hernia, hence we give none. In the language of a quaint medical writer: "Those physicians do well for all people who teach obedience to natural law, and from their ripe experience and riper wisdom, lean us against quacks and all meddlesome medication."

